# The Type of Fat in the Diet Influences the Behavior and the Relationship Between Cystinyl and Alanyl Aminopeptidase Activities in Frontal Cortex, Liver, and Plasma

**DOI:** 10.3389/fmolb.2020.00094

**Published:** 2020-05-15

**Authors:** Ana Belén Segarra, Isabel Prieto, Inmaculada Banegas, Magdalena Martínez-Cañamero, Marc de Gasparo, Patrick Vanderheyden, Stefan Zorad, Manuel Ramírez-Sánchez

**Affiliations:** ^1^Department of Health Sciences, University of Jaen, Jaen, Spain; ^2^Cardiovascular & Metabolic Syndrome Adviser, Rossemaison, Switzerland; ^3^Department of Molecular and Biochemical Pharmacology, Vrije Universiteit Brussel, Brussels, Belgium; ^4^Biomedical Research Center, Institute of Experimental Endocrinology, Slovak Academy of Sciences, Bratislava, Slovakia

**Keywords:** diet, fatty acids, cystinyl-aminopeptidase, alanyl-aminopeptidase, renin-angiotensin system

## Abstract

Insulin-regulated aminopeptidase (IRAP, cystinyl aminopeptidase, CysAP) and aminopeptidase M (alanyl aminopeptidase, AlaAP) are closely related enzymes involved in cognitive, metabolic, and cardiovascular functions. These functions may be modulated by the type of fat used in the diet. In order to analyze a possible coordinated response of both enzymes we determined simultaneously their activities in frontal cortex, liver, and plasma of adult male rats fed diets enriched with fats differing in their percentages of saturated, mono or polyunsaturated fatty acids such as sesame, sunflower, fish, olive, Iberian lard, and coconut. The systolic blood pressure, food intake, body and liver weight as well as glucose and total cholesterol levels in plasma were measured. The type of fat in the diet influences the enzymatic activities depending on the enzyme and its location. These results suggest cognitive improvement properties for diets with predominance of polyunsaturated fatty acids. Physiological parameters such as systolic blood pressure, food intake, and biochemical factors such as cholesterol and glucose in plasma were also modified depending on the type of diet, supporting beneficial properties for diets rich in mono and polyunsaturated fatty acids. Inter-tissue correlations between the analyzed parameters were also modified depending on the type of diet. If the type of fat used in the diet modifies the behavior and relationship between CysAP and AlaAP in and between frontal cortex, liver and plasma, the functions in which they are involved could also be modified.

## Introduction

**Figure 1:**
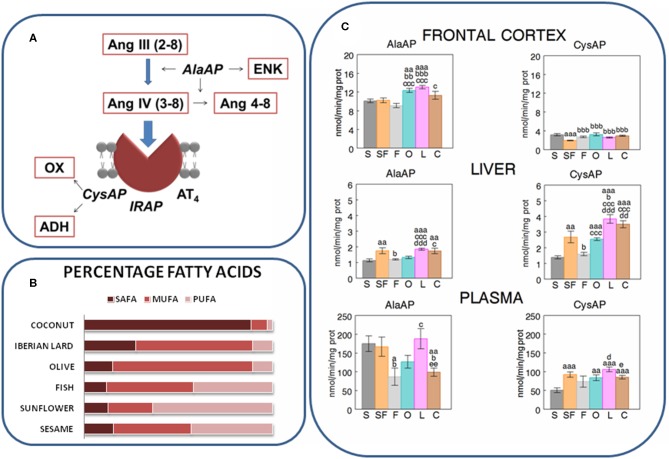
**(A)** Partial scheme of the last steps of the renin-angiotensin system in which appear the enzymatic activities measured in the present work: alanyl-aminopeptidase (AlaAP) and cystinyl-aminopeptidase (CysAP), reported to be identical to insulin-regulated aminopeptidase (IRAP) and to the AT_4_ receptor. The scheme also represents the susceptible endogenous substrates, other than angiotensins. ENK, encephalin; OX, oxytocin; ADH, antidiuretic hormone (vassopresin) (Ramírez-Sánchez et al., 2013). **(B)** Relative percentages of saturated (SAFA), monounsaturated (MUFA), and polyunsaturated (PUFA) fatty acids in the oils used in the present study to enrich the different types of diets: Respectively (SAFA, MUFA, PUFA), for sesame- (15.7, 41, 43.4), sunflower- (12, 22.4, 60.7), fish- (12, 45.8, 41.6), olive- (13.2, 73.2, 8.9), Iberian lard- (27.6, 62, 9.4), and coconut-oil (86.5, 5.8, 1.8) (Segarra et al., 2008). **(C)** Mean ± S.E.M. levels (*n* = 8) of AlaAP and CysAP activities (nmol/min/mg prot) obtained in frontal cortex, liver and plasma of male rats fed during 16 weeks with diets enriched with sesame- (S, charcoal), sunflower- (SF, rose), fish- (F, gray), olive- (O, cyan), Iberian lard- (L, magenta) and coconut-oil (C, brown). (a) indicates a significant difference in comparison with S; (b) significant difference with SF; (c) significant difference with F; (d) significant difference with O; (e) significant difference with L. Single letter, *P* < 0.05; double letter, *P* < 0.01; triple letter, *P* < 0.001.

Ang III at the final steps of the cascade of the renin-angiotensin system (Figure 1A) is metabolized to Ang IV, through the action of alanyl aminopeptidase (AlaAP, EC. 3.4.11.2). Ang IV is further metabolized to Ang 4-8 by the action of AlaAP. By its binding to the AT_4_ receptor, identified as insulin-regulated aminopeptidase (IRAP or cystinyl aminopeptidase, CysAP, EC. 3.4.11.3, AT_4_), Ang IV may be involved in cognitive and cardiovascular functions as well as in glucose metabolism. Its role in glucose uptake in brain is region-specific and dependent on a high colocalization of IRAP and the glucose transporter GLUT4 (Fernando et al., 2008). However, the effects of Ang IV on glucose uptake and the IRAP function may be independent (De Bundel et al., 2009). On the other hand, AlaAP may also hydrolyze enkephalins whereas CysAP hydrolyzes oxytocin and vasopressin (reviewed in Ramírez-Sánchez et al., 2013). The inhibition of both AlaAP (Ismail et al., 2017) and IRAP (Diwakarla et al., 2016; Seyer et al., 2019) have been proposed as strategies to improve cognitive functions such as memory processes. Therefore, both AlaAP and CysAP activities might act in concert to affect the cognitive, cardiovascular and metabolic functions in which they have been involved.

The type of fat in the diet modifies the profile of fatty acids and the levels of certain neuropeptidase activities in frontal cortex (Segarra et al., 2011, 2019a) as well as the levels of cholesterol in plasma (Segarra et al., 2008). In addition, the type of fat in the diet affects the correlation between some neuropeptidase activities and certain fatty acids in frontal cortex (Segarra et al., 2011, 2019a). Changes of the fatty acids profile in cells may affect the fluidity of membranes and consequently the association between the enzyme and the membrane as well as the binding of the enzyme with its endogenous substrates (Youdim et al., 2000; Segarra et al., 2011). Therefore, if the proteolytic activities change, the levels and functions in which their endogenous substrates are involved, such as cognitive functions, may also be modified.

Furthermore, different types of diets affect the metabolism of lipids, carbohydrates, and proteins on target organs such as the liver particularly involved in glucose metabolism, where they can influence glucose levels and fatty acids, and the brain, where they can modulate cognitive processes. The study of the effect of various saturated and unsaturated fats in the diet should give us an overview of the behavior of neuropeptidases and therefore, on the functional status of their endogenous substrates. These effects on the hepatic, cerebral and plasma response of such enzymes could be produced through a gut-brain-liver direct axis (Wang et al., 2008) or by indirect action linked to the induction of the development of different types of microbiota (Mayer et al., 2015; Martínez et al., 2019).

Tissues do not function as independent compartments: they interact among each other to offer an integrated response to changes in the internal and/or external environment (Samdani et al., 2015; Segarra et al., 2019b). If the level of activity of each enzyme in each tissue can be differentially modified depending on the type of diet, a possible interaction between such intra and inter-tissue activities could also be modified. In order to contrast the influence of the degree of saturation of fat on the diet (Figure 1B) and to better understand a neurovisceral integrative response to the effect of specific diets, it is necessary not only to determinate the profile of the response to selective diets at different locations but also to obtain information on the intra- and inter-tissue interactions between these locations (Samdani et al., 2015) and enzymes. Therefore, AlaAP and CysAP activities were determined in frontal cortex (FC), liver (LI), and plasma (PL) of male rats fed diets enriched with sesame oil (S), sunflower oil (SF), fish oil (F), olive oil (O), Iberian lard (L), or coconut oil (C). Systolic blood pressure (SBP), food intake (FI), body weight (BW), liver weight (LW), as well as total cholesterol (TCH) and glucose (GLU) levels in plasma were also measured.

## Materials and Methods

Forty eight adult male Wistar rats, weighing 200–250 g (aged 3–4 months) at the beginning of the study, were divided in six groups (*n* = 8 each), individually housed in metabolic cages and kept under standard environmental conditions. To ensure a full effect of the diets on experimental animals and based on the average length of the diet used in the literature, each group was fed during 16 weeks with isocaloric diets supplemented with 10% of the different oils studied: S, SF, F, O, L, and C (Segarra et al., 2008). At the end of the feeding period, the rats were weighed and their systolic blood pressure recorded by plethysmography (Segarra et al., 2008). Under equithesin anesthesia, blood samples were obtained from the left cardiac ventricle and then, the animals were totally perfused with saline. Their brains were quickly removed and cooled in dry ice. The livers were removed, weighed and samples from the left lateral lobe were obtained and cooled in dry ice. From brains, the frontal lobes (11.20 mm anterior to the interaural line) were dissected according to the stereotaxic atlas of Paxinos and Watson (1998). Plasma was isolated by centrifugation of blood samples for 10 min at 2,000 g using heparin as an anticoagulant and stored at −20°C. TCH and GLU were determined colourimetrically in plasma using kits supplied, respectively, by Sigma (St Louis, MO) and Spinreact (Girona, Spain) and expressed as mg/dL (Segarra et al., 2019b). In order to avoid its possible influence on the studied factors such as CysAP (Habtemichael et al., 2015), fasting conditions were not included in the study. Although it has been reported the influence of equithesin anesthesia on glucose levels (Bola and Kiyatkin, 2016), all groups were treated in the same conditions, which permit to discriminate the effects of the different diets on the studied factors. All experimental procedures were in accordance with the European Communities Council Directive 86/609/EEC. Enzymatic assays were performed as previously described Ramírez et al. (2011). Briefly, brain and liver samples were homogenized in 400 μl of 10 mM HCl-Tris buffer (pH 7.4) and ultracentrifuged at 100,000 × g for 30 min at 4°C. To obtain the particulate fraction, the pellets were re-homogenized in HCl-Tris buffer (pH 7.4) plus 1% Triton-X-100. After centrifugation (100,000 × g, 30 min, 4°C), the supernatants were shaken in an orbital rotor for 2 h at 4°C with the polymeric adsorbent Bio-Beads SM-2 (100 mg/ml) to remove the detergent from the sample. After bio-beads removal, these supernatants were used to measure AlaAP and CysAP activities as well as protein content in triplicate (Ramírez et al., 2011). AlaAP and CysAP levels were measured using Ala- or Cys-β-naphthylamide as substrates. Ten microlitre of each supernatant and plasma were incubated for 30 min at 25°C with 1 ml of the substrate solution, i.e., 2.14 mg/100 ml of Ala-β-naphthylamide or 5.53 mg/100 ml of Cys-β-naphthylamide, 10 mg/100 ml BSA, and 10 mg/ 100 ml DTT in 50 mM of phosphate buffer (pH 7.4 for AlaAP) and 50 mM HCl-Tris buffer (pH 6 for CysAP). The reactions were terminated by the addition of 1 ml of 0.1 mol/l of acetate buffer, pH 4.2. The amount of -naphthylamine released as a result of the enzymatic activity was measured fluorometrically at a 412 nm emission wavelength with an excitation wavelength of 345 nm. Proteins were quantified in triplicate (Bradford, 1976) with BSA as a standard. Specific activities were expressed as pmol of the corresponding substrate hydrolyzed per min per mg of protein. Fluorogenic assays were linear with respect to time of hydrolysis and protein content. For statistical analysis, to analyze differences between groups, one-way analysis of variance (ANOVA) was used. *Post-hoc* comparisons were made using the Student's *t*-test. Pearson's coefficient of correlation was computed to study the possible intra- and inter-tissue association of the parameters studied. Computations were performed using SPSS 13.0 and STATA 9.0. P-values below 0.05 were considered significant.

## Results

**Figure 2:**
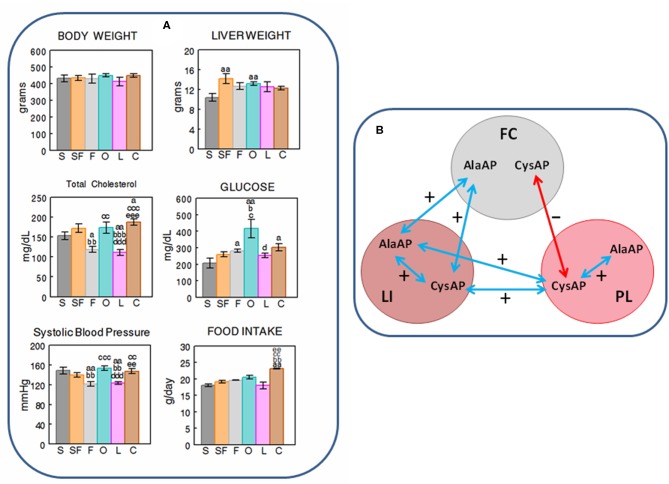
**(A)** Mean ± S.E.M. levels (*n* = 8) of body and liver weight (grams), total cholesterol and glucose levels in plasma (mg/dL), systolic blood pressure levels (mmHG) and food intake (g/day), obtained at the end of the feeding period in male rats fed during 16 weeks with diets enriched with sesame- (S, charcoal), sunflower- (SF, rose), fish- (F, gray), olive- (O, cyan), Iberian lard- (L, magenta), and coconut-oil (C, brown). (a) indicates a significant difference in comparison with S; (b) significant difference with SF; (c) significant difference with F; (d) significant difference with O; (e) significant difference with L. Single letter, *P* < 0.05; double letter, *P* < 0.01; triple letter, *P* < 0.001. **(B)** Simplified scheme showing the intra- and inter-tissue significant correlations between the enzymatic activities. Blue arrows denote positive correlations. Red arrow denotes negative correlation. FC, frontal cortex; LI, liver; PL, plasma.

The results are indicated in Figures 1, 2 and in Tables 1, 2. The levels of TCH and SBP (Segarra et al., 2008) as well as AlaAP activity with F, O and C diets (Segarra et al., 2019a) were previously reported. In *frontal cortex*, O and L diets exhibited higher levels of AlaAP activity than after the S diet, SF and F diets (*p* < 0.01 and *p* < 0.001) and C diet induced higher levels of AlaAP activity than F diet (*p* < 0.05). CysAP had lower activity with the SF diet than with the other diets (*p* < 0.001). In contrast, C diet demonstrated higher CysAP activity levels than the F diet (*p* < 0.05). In *liver*, the S diet caused lower levels of AlaAP activity than the SF (*p* < 0.01), L (*p* < 0.001), and C (*p* < 0.01) diets. Further, L had higher levels of AlaAP activity than S, F and O (*p* < 0.001). C was also higher than S (*p* < 0.01) and F (*p* < 0.05) for AlaAP activity. In liver, CysAP had higher levels of activity with L diet than with S (*p* < 0.001), SF (*p* < 0.05), F (*p* < 0.001), and O diet (*p* < 0.001) whereas C was higher than S (*p* < 0.001), F (*p* < 0.001) and O (*p* < 0.01) diets and S was lower than SF (*p* < 0.01), O, L, and C (*p* < 0.001). In *plasma*, AlaAP activity was lower with F than with S, SF, and L diets (*p* < 0.05), C was also lower than S (*p* < 0.01), SF (*p* < 0.05), and L (*p* < 0.01) diets. In plasma, CysAP activity was also lower with S diet than with SF (*p* < 0.001), O (*p* < 0.01), L (*p* < 0.001), and C (*p* < 0.001) diets. O and C diets had lower levels of CysAP activity than L (*p* < 0.05) (Figure 1C).

**Table 1:** Intra- and inter-tissue significant correlations (paired data) of the different parameters measured into and between the tissues analyzed: Frontal cortex (FC), liver (LI), and plasma (PL) in each one of the diets studied (*n* = 8).

**Sesame**			**Sunflower**			**Fish**		
**Correlation**	***r***	***P***	**Correlation**	***r***	***P***	**Correlation**	***r***	***P***
FC CysAP vs. PL AlaAP	−0.772	0.02	LI AlaAP vs. LI CysAP	+0.881	0.003	LI AlaAP vs. LI CysAP	+0.775	0.02
FC CysAP vs. PL CysAP	−0.798	0.01				PL AlaAP vs. PL CysAP	+0.935	0.0006
PL AlaAP vs. PL CysAP	+0.816	0.01						
SBP vs. Glucose	−0.777	0.02						
**Olive**			**Iberian lard**			**Coconut**		
FC CysAP vs. LI AlaAP	+0.708	0.04	FC AlaAP vs. FC CysAP	+0.795	0.01	No correlations		
FC AlaAP vs. FC CysAP	+0.884	0.003	LI AlaAP vs. LI CysAP	+0.813	0.01			
Glucose vs. LI CysAP	−0.767	0.02	SBP vs. Glucose	+0.803	0.01			

*Negative correlations with rose background. Positive correlations with blue background. The values of r and P are indicated*.

**Table 2:** Values of r for significant correlations (paired data) obtained between the different physiological and biochemical parameters determined at each location (Frontal cortex, FC; Liver, LI; Plasma, PL), considering together all the data (*n* = 48) obtained with the six groups designed (sesame, sunflower, fish, olive, Iberian lard, coconut).

	**Body wt**	**Liver wt**	**SBP**	**FI**	**Total Ch**	**Glucose**	**FC AlaAP**	**FC CysAP**	**LI AlaAP**	**LI CysAP**	**PL AlaAP**	**PL CysAP**
Body wt	1	+0.642			+0.359				+0.290			+0.352
Liver wt		1						−0.312	+0.391			+0.560
SBP			1		+0.518							
FI				1								
Total Ch					1							
Glucose						1						
FC AlaAP							1		+0.425	+0.575		
FC CysAP								1				−0.389
LI AlaAP									1	+0.803		+0.366
LI CysAP										1		+0.464
PL AlaAP											1	+0.469
PL CysAP												1

*Blue background for positive values and rose background for negative ones*.

While weight gain (data not shown) and body weight demonstrated no differences between the different types of diets at the end of the feeding period, the liver weight showed to have lower levels with the S diet than with SF and O diets(*p* < 0.01). Total cholesterol in plasma demonstrated lower levels with F and L diets than with the rest of diets, and glucose was higher with O than with S, SF, F and L diets. The S diet had lower levels of plasma glucose than F, O and C diets. Systolic blood pressure exhibited the same profile than TCH: lower levels of SBP with F and L diets than with the rest. FI was higher (*p* < 0.01) with the C diet than with the rest of diets except with O (Figure 2A).

When we analyzed the data for the search of intra- and inter-tissue correlations with each of the diets studied, significant *inter-tissue* correlations between enzymatic activities were only obtained with S (between FC vs. PL: FC CysAP vs. PL AlaAP and FC CysAP vs. PL CysAP) and with O (between FC vs. LI: FC CysAP vs. LI AlaAP). There were negative correlations with S and positive with O. SBP correlates negatively with GLU in the diet enriched with S and positively in the diet enriched with L. Glucose also correlates negatively with CysAP activity from LI in the O diet. *Intra-tissue* correlations between both enzymatic activities were observed in FC with O and L diets, in LI with SF, L and F diets and in PL with the F diet. No significant correlations were observed in the C diet (Table 1).

Considering together the data obtained with the six diets studied (*n* = 48), we observed significant positive correlations between BW vs. LW and TCH but also with LI AlaAP and PL CysAP activities. LW correlates negatively with FC CysAP and positively with LI AlaAP and PL CysAP activities. SBP correlates positively with TCH. While FC AlaAP correlates positively with LI AlaAP and LI CysAP activities, FC CysAP correlates negatively with PL CysAP activity. LI AlaAP correlates positively with LI CysAP and PL CysAP activities, and PL CysAP correlates positively with LI CysAP and with PL AlaAP activities (Table 2 and Figure 2B).

## Discussion

AlaAP and CysAP activities are involved in the metabolism of enkephalins, oxytocin and vasopressin as well as in the glucose metabolism. Changes in enzyme activities depending on the type of fat in the diet may be related to changes in the functions they exert in the locations studied: FC, LI, and PL. Consequently we could expect modulations in cognitive and metabolic functions in which these enzymes are involved (Ramírez-Sánchez et al., 2013). However, since we have not included a parallel study using specific IRAP inhibitors, the CysAP activity measured in the present work using an arylamide derivative as substrate does not necessarily corresponds to IRAP activity and therefore the interpretation of the results should be considered with caution.

It has been reported that the AT_4_ receptor was identified as IRAP being also co-localized with the glucose transporter GLUT4. It was proposed that the binding of Ang IV to AT_4_ results in the inhibition of its enzymatic activity, reducing the catabolism of their endogenous substrates (vasopressin, oxytocin) and consequently increasing their availability and extending their action. Therefore, through its high affinity binding to the AT_4_ receptor, Ang IV might modulate cognitive and metabolic functions via neuropeptide processing or local blood flows. In this context, since AlaAP activity is involved in Ang III and Ang IV metabolism and it also acts as enkephalinase, this enzyme may as well be involved in these cognitive and metabolic regulations (Ramírez-Sánchez et al., 2013; Ismail et al., 2017).

In FC, the high difference between diets for AlaAP and CysAP activities suggests a role for the type of fatty acids on these enzymatic activities and therefore on their substrates. For example the low levels of both activities with SF (rich in polyunsaturated fatty acids) may suggest longer action for the AlaAP substrates enkephalins and Ang III and a longer action for the CysAP substrate oxytocin. Polyunsaturated fatty acids enriched diet has been proposed to improve cognitive functions (Chalon et al., 2001). Enkephalins (Henry et al., 2017), and oxytocin (Wagner and Echterhoff, 2018) were also reported as cognitive improving neuropeptides. If a diet enriched in polyunsaturated fatty acids results in reduced levels of enkephalinase and oxytocinase activities in comparison with other diets, both neuropeptides may prolong their beneficial effects in frontal cortex. As previously indicated, the specific fatty acids membrane pattern induced by this type of diet may affect membrane fluidity and the association with the membrane-bound enzyme and so influence the hydrolytic capability of the enzyme (Youdim et al., 2000; Segarra et al., 2011). These results are compatible with an improvement of cognitive functions such as memory processes (Diwakarla et al., 2016; Seyer et al., 2019) for enriched diets in polyunsaturated fatty acids such as SF. In LI, with S and F (rich in mono and polyunsaturated fatty acids) there were lower levels of AlaAP and CysAP activities which may suggest an increase in local blood flow. In PL, for example, there was a clear difference between AlaAP and CysAP with the S diet: whereas AlaAP exhibited high activity levels (suggesting high metabolism of Ang III, Ang IV and enkephalins), CysAP had low levels of activity, suggesting longer action of vasopressin.

With the diet enriched with O, there was a negative correlation between GLU and CysAP activity in liver: The lower CysAP activity in liver, the higher GLU in PL and vice versa. This could be interpreted as a direct influence of the O diet on the glucose transporter and/or a compensatory response of CysAP in liver to the increase in plasma of GLU (Figure 2B). Other authors have described diverse influences of diets on plasma glucose. For example, Buettner et al. (2006), analyzing diets enriched with lard, olive, coconut and fish oil reported high levels of plasma glucose in olive, in comparison with the rest of diets. Dulloo et al. (1995), studying diets enriched with lard, coconut, olive, sunflower and fish did not observe differences for glucose levels between diets. Giron et al. (1999) reported lower glucose levels in plasma of animals fed a diet enriched with fish oil than the ones enriched with olive and sunflower oils. Interestingly, it has been discovered that upper intestinal lipids activate a gut-brain-liver axis that regulates liver glucose homeostasis (Wang et al., 2008). In addition, hypercholesterolemia has been associated with cognitive disorders linked to GLUT4 expression and modulated by CysAP and aminopeptidase N activities (Ismail et al., 2017). Our results are therefore in agreement with these observation: changes in the levels of cholesterol, depending on the type of diet, may influence the cognitive processes. In this sense, the low levels of TCH and SBP obtained with diets enriched with F or L (Figure 2A) suggest a beneficial role for mono and polyunsaturated fatty acids.

Interestingly, with a diet enriched with S (rich in monounsaturated and polyunsaturated fat), there was a negative correlation between SBP vs. GLU but in contrast, with L (rich mainly in monounsaturated fat) this correlation was positive which supports the importance of the type of diet in the adjustment of physiologic processes. Furthermore, both if we consider individually the type of diet (S and O) (Table 1), and if we consider together the data of all diets (Table 2), the relationship of FC with PL was *negative* while that of FC with LI was *positive*, just as it is also *positive* between LI and PL. In particular, considering only the S-enriched diet (Table 1), FC CysAP correlated *negatively* with PL AlaAP and PL CysAP and also *negatively* with PL CysAP considering all data (Table 2). In contrast, FC CysAP correlated *positively* with LI AlaAP and LI CysAP with an O-enriched diet (Table 1) and FC AlaAP correlated *positively* with LI AlaAP and LI CysAP (Table 2). In addition, AlaAP and CysAP from LI, correlated *positively* with AlaAP and CysAP from PL (Table 2). From a general perspective (Figure 2B), we observed some form of *positive* feedback between FC AlaAP with LI AlaAP and CysAP, a *positive* feedback between LI AlaAP and CysAP with PL but a *negative* feedback between FC CysAP with PL CysAP. Also, while in LI and PL there was a *positive* relationship between AlaAP and CysAP, there was no correlation in FC. Whether such changes relate to the amount of enzyme present (Vmax) or the conformation of the enzyme (Km) remains to be analyzed.

As we could expect, the results demonstrated significant positive correlations for BW vs. LW and vs. TCH as well as for SBP vs. TCH (Table 2). These results support the validity of our observation. In conclusion, the present results apparently do not show a clear systematic profile of response depending on the type of diet but they could be considered as preliminary results which support a distinctive influence of the saturation of the fatty acids in the diet that may result in changes in cognitive and metabolic functions which deserve further specific research.

## Data Availability Statement

The raw data supporting the conclusions of this article will be made available to any qualified researcher.

## Ethics Statement

The animal study was reviewed and approved by Ethics committee of the University of JAÉN.

## Author Contributions

AS and IP contributed equally to the work with the acquisition, analysis, and interpretation of data. MR-S contributed to the acquisition, analysis and interpretation of data and wrote the first manuscript draft. MM-C, IB, PV, SZ, and MG participated in the analysis and interpretation of data and revised critically the final form of the manuscript. All authors have approved the final manuscript.

## Conflict of Interest

The authors declare that the research was conducted in the absence of any commercial or financial relationships that could be construed as a potential conflict of interest.
